# Equine Rhinosporidiosis in United Kingdom

**DOI:** 10.3201/eid1309.070532

**Published:** 2007-09

**Authors:** Gail Leeming, Ken C. Smith, Mark E. Bestbier, Annalisa Barrelet, Anja Kipar

**Affiliations:** * University of Liverpool, Liverpool, United Kingdom; † Animal Health Trust, Newmarket, Suffolk, United Kingdom; ‡ Beaufort Cottage Laboratories, Newmarket, Suffolk, United Kingdom; 1 Current affiliation: The Royal Veterinary College, Hatfield, United Kingdom; 2 Current affiliation: Rest Associates, Swaffham Prior, Cambridge, United Kingdom

**Keywords:** rhinosporidiosis, horse, hyperplastic rhinitis, emerging disease, dispatch

## Abstract

We report 4 cases of equine rhinosporidiosis in the United Kingdom. These cases provide evidence of spread of infectious agents from rhinosporidiosis-endemic areas to nonendemic areas by increased international movement of livestock. Surveillance should continue for this infective agent of potential relevance for numerous species, including humans.

Rhinosporidiosis is caused by *Rhinosporidium seeberi*, an organism that was previously classified as a fungus but has been regrouped into the class Mesomycetozoa (family Rhinosporideacae). This class consists of several parasitic and saprophytic organisms, most of which infect fish and amphibians; only *R. seeberi* infects mammals (*1*,*2*). Rhinosporidiosis is endemic to India and Sri Lanka, although cases have been reported in Africa, the Americas, and Europe (*3*). Most affected patients have a history of temporary or permanent residence within rhinosporidiosis-endemic areas. Rhinosporidiosis is predominantly a human disease; however, it has been documented in many other species, including cats, dogs, cattle, and waterfowl (*4*). Equine cases are infrequent but have been reported from the southern United States (*5*), South America (*6*), and South Africa (*7*). The first equine case (1 of those detailed in this article) in the United Kingdom was recently reported (*8*).

The natural habitat of *R. seeberi* is thought to be stagnant or lacustrine water, although isolation of the organism from such environments has so far been unsuccessful (*9*). Nonetheless, epidemiologic evidence supports this hypothesis; the only report of an outbreak originating within Europe was associated with persons bathing in a lake in Serbia (*10*). Because the typical location of *R. seeberi*–associated lesions in all species is the nasal mucosa, drinking from contaminated water is likely the source of infection (*11*), possibly through superficial wounds in the mucosa. In addition, for ocular disease, dust particles are possible fomites for endospores (*4*). Rhinosporidiosis commonly causes single or multiple, sessile or pedunculated, papillomatous, polypoidal or compact masses within the nasal mucosa or, less frequently, the ocular mucosa. These masses are painless, slow-growing, and noninfiltrating. Surgical excision is the treatment of choice (*4*).

## The Study

We describe 4 cases of rhinosporidiosis in polo ponies imported into the United Kingdom from Argentina and kept in different locations. Diagnoses were made over a 6-month period by routine histopathologic examination at 3 diagnostic centers. For confirmation of the causative agent, DNA was extracted from biopsy samples and skin of an unaffected horse (negative control) by using a commercially available kit (DNeasy Tissue Kit, QIAGEN Ltd., Crawley, West Sussex, UK) according to the manufacturer’s protocol. *R. seeberi–*specific primers for the 18S rDNA sequence (*3*) were used in a PCR, and the *R. seeberi–*specific amplification product from 1 sample was sequenced by Lark Technologies (Takeley, Essex, UK), as described (*8*).

**Figure 1:**
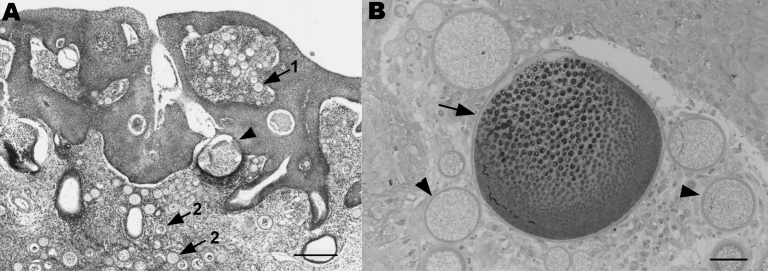
A) Section of nasal mucosa showing multifocal hyperplasia, juvenile sporangia (arrows) within the mucosal epithelium (1) and the lamina propria mucosae (2), and mature sporangia (arrowhead). A multifocal mixed inflammatory infiltrate can be seen within the mucosa. Stain, hematoxylin and eosin; magnification ×4; scale bar, 250 μm. B) Semithin section of nasal mucosa with juvenile sporangia (arrowheads) and a mature sporangium (arrow) with a lymphoplasmacellular inflammatory infiltrate within the lamina propria mucosae. Stain, toluidine blue; magnification ×10; scale bar, 40 μm.

The ponies had clinical signs such as epistaxis, or they had been asymptomatic and a lesion was noticed during routine examination. One pony had a clinical history of epistaxis that first occurred 10 months after the animal had been imported. On gross examination, friable soft tissue masses, located unilaterally or bilaterally within the nasal mucosa, were observed. For all 4 ponies, histologic examination showed moderate multifocal hyperplasia and ulceration of the mucosa. Within the expanded mucosa, and particularly within the lamina propria mucosae, multiple spherical to polygonal organisms of variable appearance, consistent with *R. seeberi,* were seen (*6*). The smaller (<100 μm in diameter) structures had an eosinophilic and periodic acid Schiff–positive wall enclosing eosinophilic to basophilic fibrillar material (juvenile sporangia; Figure 1, panel B). The larger (<300 μm in diameter), spherical to polygonal structures had a thin eosinophilic wall with closely opposed basophilic stippled material and basophilic and eosinophilic ovoid structures (endospores) within the central lumen (mature sporangia; Figure 1, panel A). Surrounding these sporangia was a mild to moderate, multifocal, lymphoplasmacellular inflammatory infiltrate (Figure 1, panel B). Additionally, mild to marked multifocal pyogranulomatous infiltrates, most commonly associated with free endospores from ruptured mature sporangia, were noted. Mild hyperemia, mild multifocal hemorrhage, and mild multifocal hemosiderosis were also present.

**Figure 2:**
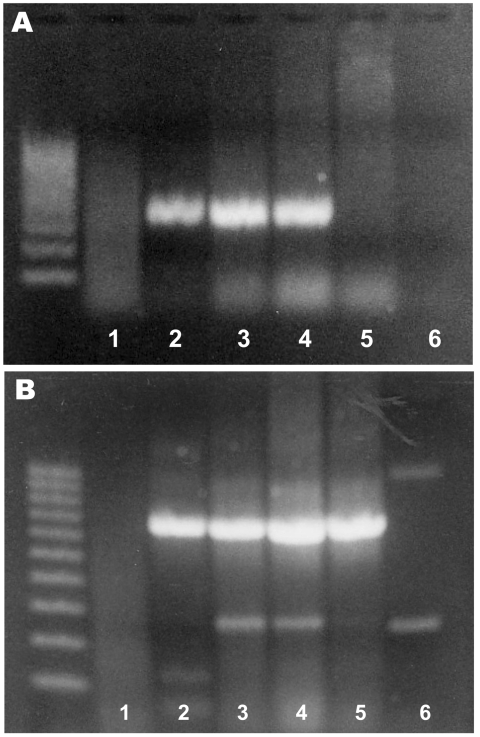
Agarose gel electrophoresis of PCR products from *Rhinosporidium seeberi*–specific primers (A) and β-actin primers (B). The left lane contains a 100-bp ladder. Samples 1–4, from horses with histologic diagnoses of rhinosporidiosis; sample 5, from the skin of a noninfected horse; sample 6, negative control (water).

PCR amplification using *R. seeberi–*specific primers provided bands of the expected size (377 bp [*3*]) in 3 of the 4 samples. PCR with primers for the housekeeping β-actin gene produced bands of the expected size in the same 3 samples and the noninfected control (Figure 2). Sequencing of 1 product was consistent with the published sequence for *R. seeberi* (*1*,*3*,*8*).

## Conclusions

This report describes what we believe to be the first veterinary cases of rhinosporidiosis in the United Kingdom. The 4 affected polo ponies had all been imported from Argentina. Rhinosporidiosis is endemic throughout India and Sri Lanka, and disease-endemic foci are found in Uganda, the United States (Texas), Brazil, and Argentina (*4*). Within Argentina, rhinosporidiosis-endemic areas include the Rio Parana and the Rio de la Plata (*12*). Unfortunately, we could not obtain information about where in Argentina the affected polo ponies originated.

The morphologic features of the agent seen in all cases presented here are characteristic, and diagnostic, for *R. seeberi* (*13*). However, potential differential diagnoses that should be considered include polypoid or granulomatous rhinitis caused by fungal infection with *Coccidioides immitis* or *Chrysosporium parvum* (the causative agent of adiaspiromycosis) and neoplasia (*11*,*13*). The result of the *R. seeberi–*specific PCR and the sequencing of 1 of the amplification products provides definitive proof that *R. seeberi* was the infective organism in 3 of the 4 samples. Because the fourth sample did not yield a product with the *R. seeberi–*specific primers and also did not yield an amplification product in a PCR for the housekeeping β-actin gene, DNA extraction was likely not successful for this sample. Such extraction failure could result from prolonged formalin fixation and paraffin embedding because these techniques can have a profound effect on the molecular arrangement of DNA and may inhibit its amplification and extraction (*14*).

The treatment of choice for rhinosporidiosis is surgical excision of lesions. In humans, lesions have been found to recur after surgery in 11% of cases, possibly because of incomplete excision or intraoperative contamination of adjacent surfaces with endospores (*4*). To prevent recurrence, electrocauterization at the site of excision is recommended. Of the ponies in this study for which the outcome is known, excision was curative for 1, but recurrence of clinical signs has occurred in another, most likely due to incomplete excision. Pharmacologic treatment has not been successful, probably because of the impenetrability of the sporangial wall (*4*).

International movement of horses, particularly for competition, is now commonplace. Such travel increases risk for exposure to diseases and pathogens not usually encountered in the importing country. With regard to the United Kingdom, ≈1,000 polo ponies are imported from Argentina every year (A. Wardall, pers. comm.), and other ponies come from New Zealand, Australia, and the United States. Thus, a relatively large number of potentially exposed ponies are imported into the United Kingdom each year. For *R. seeberi*, the possibility of a prolonged incubation period before development of clinical signs (epistaxis in 1 pony reported here did not occur until 10 months after importation) could lead to introduction of this infective agent, because lesions may not be apparent at the time of importation. Direct transmission of *R. seeberi* between humans and animals has not been proven (*4*); furthermore, multiple host-specific strains may exist (*15*). However, because an outbreak was connected with a single body of water in Europe (*10*), infected animals imported into non–rhinosporidiosis-endemic areas may contaminate such water and lead to further, autochthonous outbreaks.
